# Supplementary material for: Authentication of Shenqi Fuzheng Injection via UPLC-Coupled Ion Mobility—Mass Spectrometry and Chemometrics with Kendrick Mass Defect Filter Data Mining
Source: Molecules. 2022 Jul 24;27(15):4734. doi: 10.3390/molecules27154734 (PMC9330873; doi:10.3390/molecules27154734)
Supplement: Supplementary file 1 [file molecules-27-04734-s001.zip › molecules-1819622-supplementary.pdf]

Authentication of Shenqi Fuzheng Injection by UHPLC hyphenated ion mobility - mass spectrometry and chemometrics with Kendrick mass defect filter data mining

Xingdong Wu [1,†], Yaowen Liu [3,1,†], Zijia Zhang [1], Zhihuang Ou [4,5], Guoxiang Wang [4,5], Tengqian Zhang [1,2] , Huali Long [1] , Min Lei [1] , Liangfeng Liu [4,5], Wenhua Huang [4,5], Jinjun Hou [1,*], Wanying Wu [1,2,*] , De-an Guo [1,2]

[1] National Engineering Research Center of TCM Standardization Technology, Shanghai Institute of Materia Medica, Chinese Academy of Sciences, Shanghai 201203, China

[2] University of Chinese Academy of Sciences, Beijing 100049, China

[3] School of Chinese Materia Medica, Nanjing University of Chinese Medicine, Nanjing 210029, China

[4] Limin Pharmaceutical Factory, Livzon Group Limited, Shaoguan, 512028, China

[5] Guangdong Corporate Key Laboratory of High-end Liquid Medicine R&D, Industrialization, Shaoguan, 512028, China.

* Correspondence: jinjun_hou@simm.ac.cn (J.H.); wanyingwu@simm.ac.cn (W.W.); Tel.: +86 21 50272789

† These authors made equal contributions to this work.

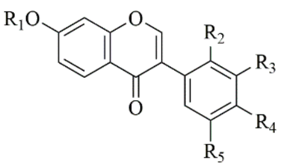

Pratensein-7-O-β-D-glucopyranoside (6): R_1=Glc, R_2=H, R_3=H, R_4=OCH_3, R5=OH

Ononin (32): R_1=Glc, R_2=H, R_3=H, R_4=OCH_3, R5=H

Calycosin-7-O-β-D-glucopyranoside (14): R_1=Glc, R_2=H, R_3=OH, R_4=OCH_3, R5=H

Calycosin (44): R_1=H, R_2=H, R_3=OH, R_4=OCH_3, R5=H

Isomucronulatol-7-O-glucoside (47): R_1=O-Glc, R_2=H, R_3=OCH3, R_4=OCH_3, R5=H

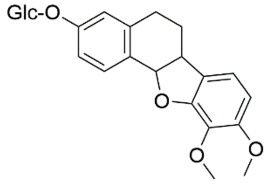

9,10-Dimethoxypterocarpan-3-O-β-D-glucoside (40)

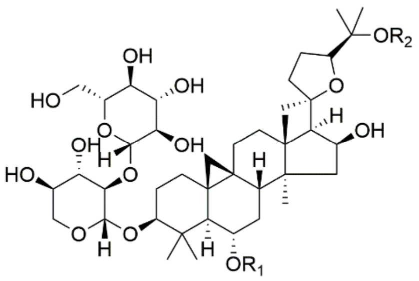

Astragaloside III(100): R1=H, R_2=H

Astragaloside VI (74): R1=Glc, R_2=H

Astragaloside V (92): R1=H, R_2=Glc

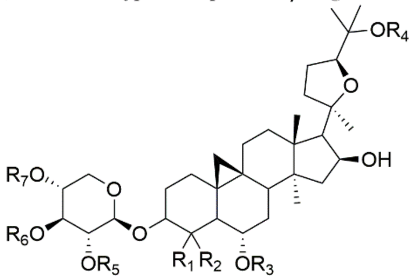

Astragaloside VII (71): R_1=CH_3, R_2=CH_3, R_3=Glc, R_4=Glc, R_5=H, R_6=H, R_7=H

Astragaloside IV (98): R_1=CH_3, R_2=CH_3, R_3=Glc, R_4=H, R_5=H, R_6=H, R_7=H

Isoastragaloside IV (101): R_1=CH_3, R_2=CH_3, R_3=H, R_4=Glc, R_5=H, R_6=H, R_7=H

Astragaloside II (105): R_1=CH_3, R_2=CH_3, R_3=Glc, R_4=H, R_5=Ac, R_6=H, R_7=H

Soyasaponin I (106): R_1=CH_3, R_2=CH_3, R_3=Glc, R_4=H, R_5=Ac, R_6=Ac, R_7=H

Isoastragaloside II (114): R_1=CH_3, R_2=CH_3, R_3=Glc, R_4=H, R_5=H, R_6=Ac, R_7=H

Cyclocephaloside II (117): R_1=CH_3, R_2=CH_3, R_3=Glc, R_4=H, R_5=H, R_6=H, R_7=Ac

Isoastragaloside I (127): R_1=CH_3, R_2=CH_3, R_3=Glc, R_4=H, R_5=Ac, R_6=H, R_7=Ac

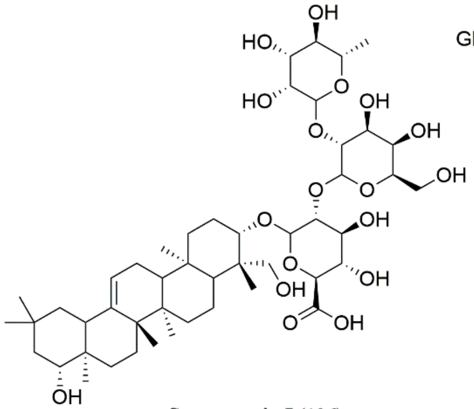

Soyasaponin I (106)

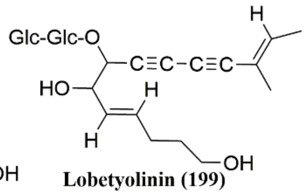

Lobetyolinin (199)

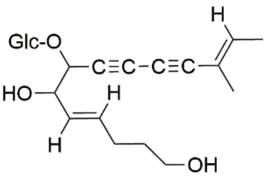

Lobetyolin (203)

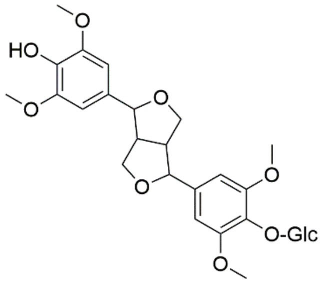

Syringaresinol 4'-O-glucopyranoside (214)

Figure. S1 Chemical structures of nine reference standards.

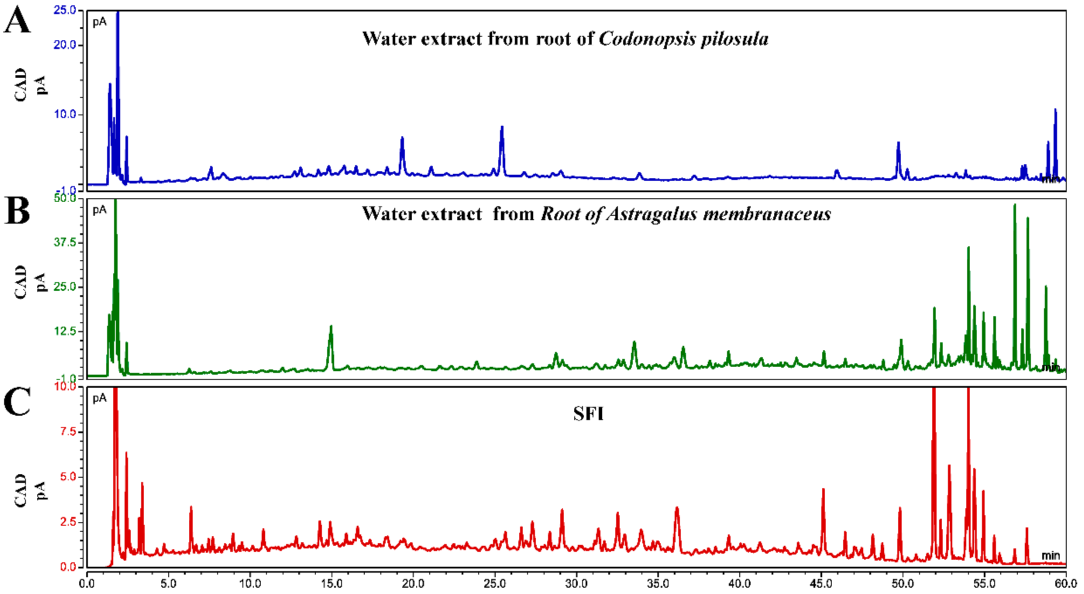

Figure. S2 Fingerprint chromatogram of UHPLC-CAD. (A: Codonopsis pilosula, B: Astragalus memeranaceus, C: SFI)

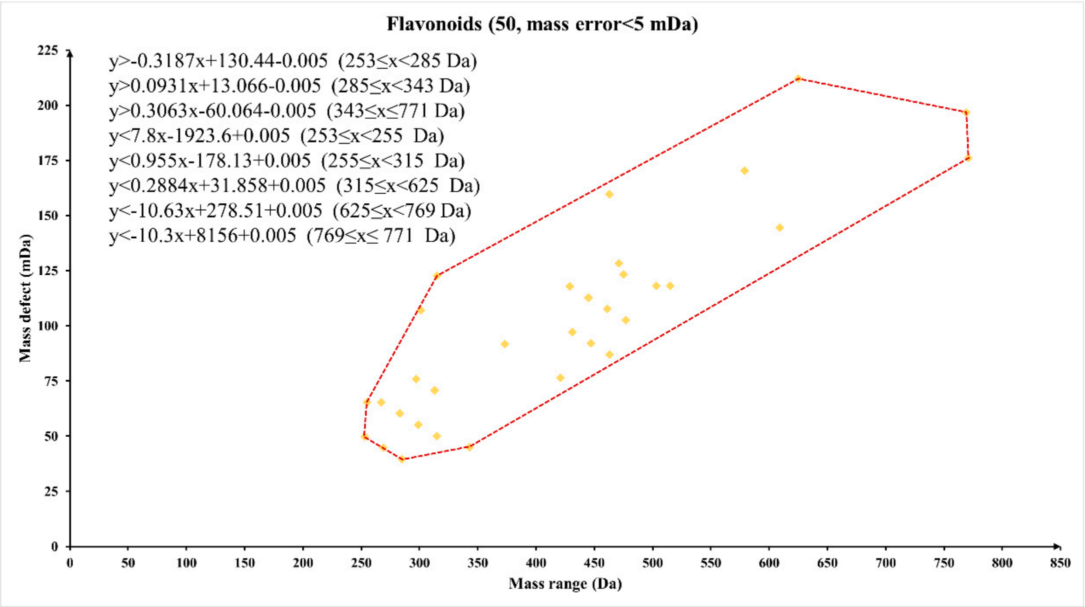

Figure. S3 Definition of PMDF windows of flavonoids through several excel formulas.

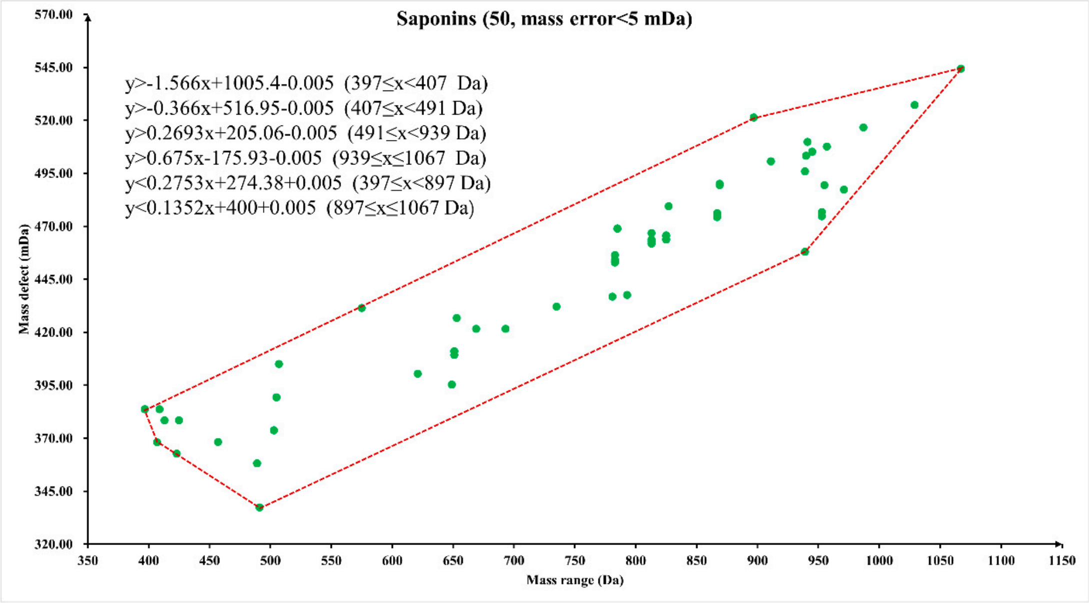

Figure. S4 Definition of PMDF windows of saponins through several excel formulas.

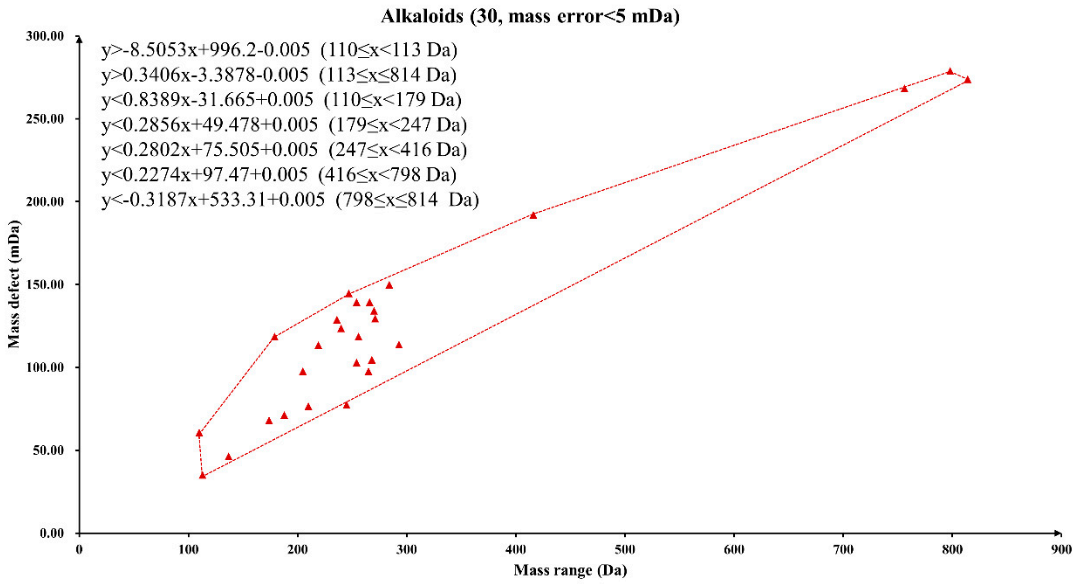

Figure. S5 Definition of PMDF windows of alkaloids through several excel formulas.

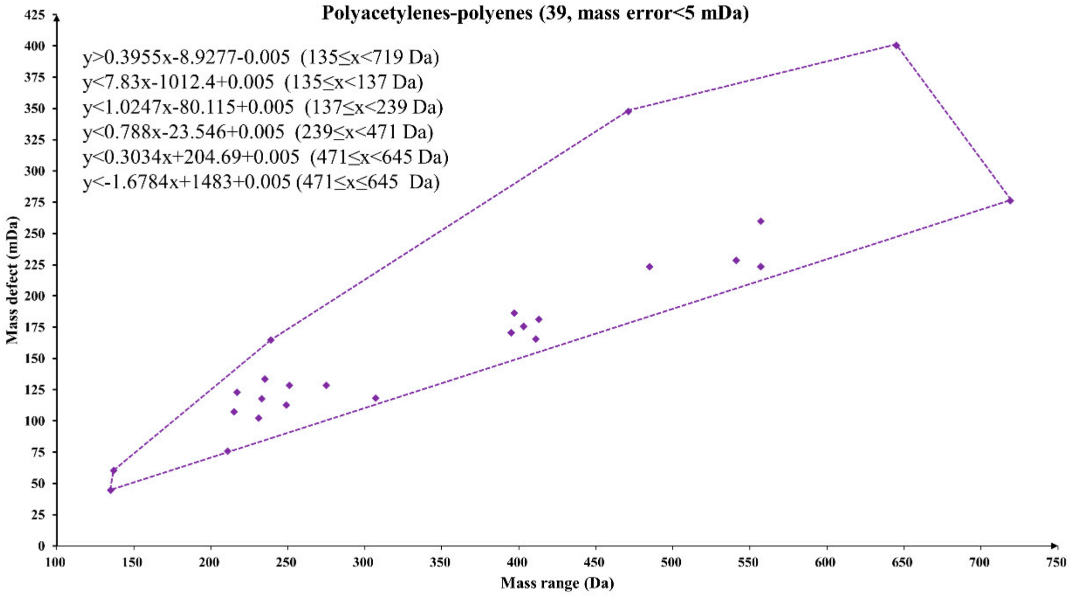

Figure. S6 Definition of PMDF windows of polyacetylenes, and polyenes through several excel formulas.

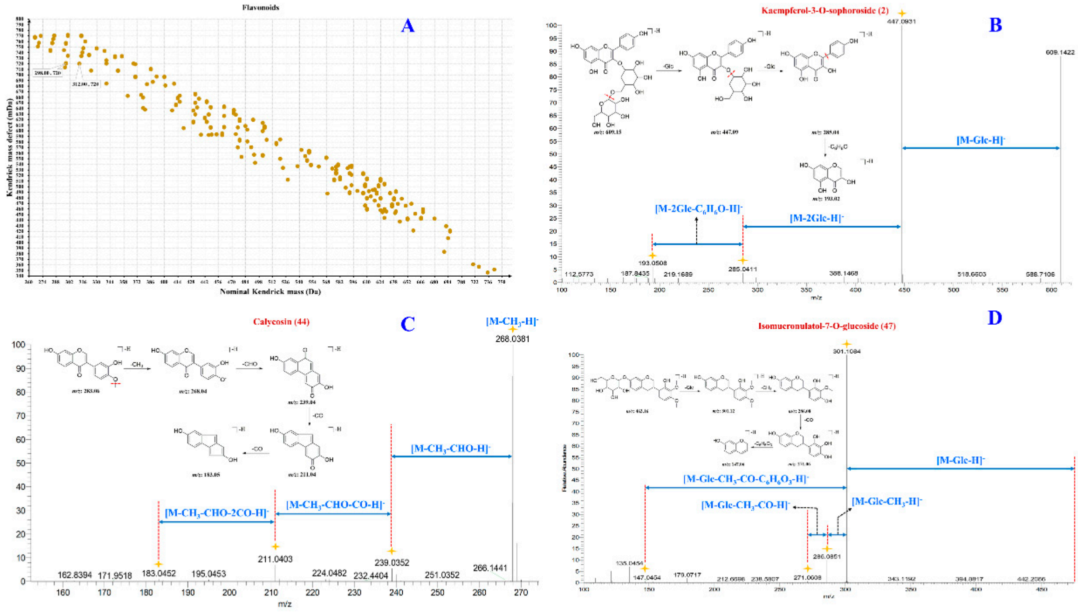

Figure. S7 KMDF scatter plot depicted the m/z values of potential flavonoid components in SFI after PMDF filtering, and the MS/MS spectra and possible MS fragment patterns of the representative flavonoids. (A: KMDF scatter plot

of potential flavonoids; B: kaempferol-3-O-sophoroside; C: calycosin; D: isomucronulatol-7-O-glucoside).

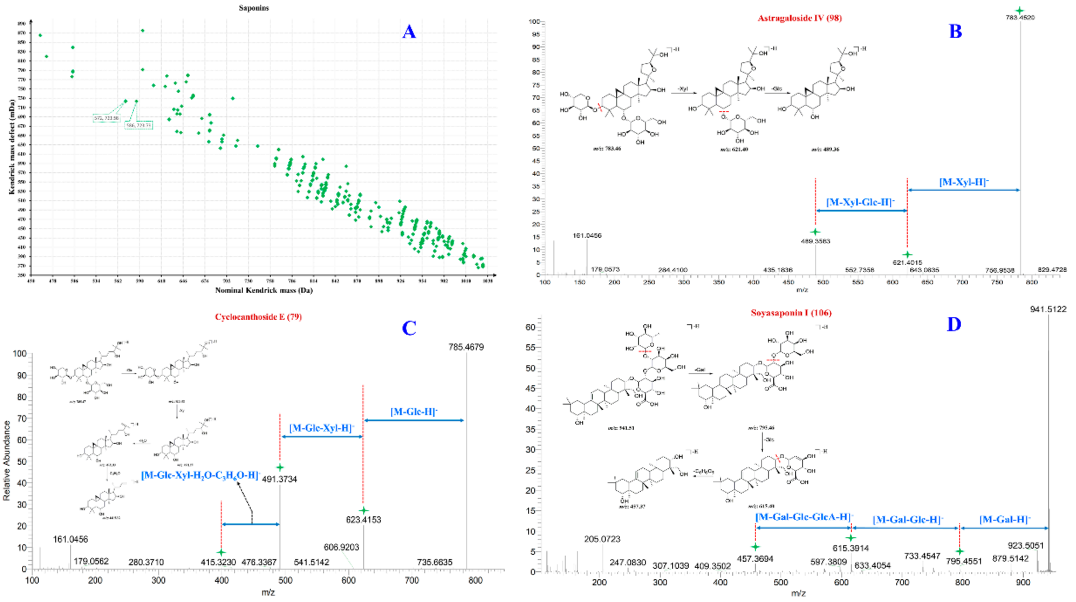

Fig. S8 KMDF scatter plot depicted the m/z values of potential saponin components in SFI after PMDF filtering, and the MS/MS spectra and possible MS fragment patterns of the representative saponins. (A: KMDF scatter plot of potential saponins; Bastragaloside IV; C: cyclocanthoside E; D: Soyasaponin I).

Table S1 The compound information used in establishing the PMDF window for flavonoids in SFI

No.	Compound name	Formula	m/z [M-H]$^-$ (Da)
1	Complanaruside	C28H32O16	623.1602
2	Formononetin	C16H12O4	267.0654
3	Formononetin-7-glucoside	C22H22O9	429.1179
4	Ononin	C25H24O12	515.1182
5	Genistin	C21H20O10	431.0972
6	Isoliquiritigenin	C15H12O4	255.0654
7	Isomucronulatol	C17H18O5	301.1071
8	Isomucronulatol-7-O-β-glucoside	C23H28O10	463.1596
9	Isomucronulatol-7-O-glucoside	C23H28O10	463.1596
10	Isomucronulatol-7-2-Di-O-glucoside	C29H38O15	625.2121
11	Isoquercitrin	C21H20O12	463.0870
12	Isorhamnetin	C16H12O7	315.0501
13	Isorhamnetin-3-O-glucoside	C22H22O12	477.1026
14	Kaempferol	C15H10O6	285.0396
15	Kumatakenin	C17H14O6	313.0708
16	Mucronulatol-7-O-glucopyranoside	C23H28O10	463.1596
17	Mangiferin	C19H18O11	421.0765
18	Mucronulatol-7-O-glucoside	C23H28O10	463.1596
19	Naringenin-7-Rhamnoglucoside	C27H32O14	579.1704
20	Naringin	C27H32O14	579.1704
21	Naringoside	C27H32O14	579.1704
22	Neocomplanoside	C24H24O12	503.1182
23	Odoratin	C17H14O6	313.0708
24	Odoratin-7-O-β-D-glucoside	C23H24O11	475.1233
25	Ononin	C22H22O9	429.1179
26	Pratensein-7-glucoside	C22H22O11	461.1077
27	Pratensein	C16H12O6	299.0552
28	Rhamnocitrin	C16H12O6	299.0552
29	Rutin	C27H30O16	609.1446

30	Sissotrin	$C_{22}H_{22}O_{10}$	445.1128
31	Ternatin	$C_{19}H_{18}O_8$	373.0918
32	Wharangin	$C_{17}H_{12}O_8$	343.0450
33	Wogonin	$C_{16}H_{12}O_5$	283.0603
34	2-Hydroxy-3-4-7-trimethoxyisoflavan	$C_{18}H_{20}O_5$	315.1227
35	3-O-Methylorobol	$C_{16}H_{12}O_6$	299.0552
36	Calycosin	$C_{16}H_{12}O_5$	283.0603
37	6-O-Acetylononin	$C_{24}H_{24}O_{10}$	471.1284
38	7-O-Methylisomucronulatol	$C_{18}H_{20}O_5$	315.1227
39	Calycosin-7-O-*β*-D-glucoside	$C_{22}H_{22}O_{10}$	445.1128
40	4-7-Dihydroxyisoflavone	$C_{15}H_{10}O_4$	253.0498
41	7-3-dihydroxy-8-4-dimethoxyisoflavone	$C_{17}H_{14}O_6$	313.0708
42	Afromosin	$C_{17}H_{14}O_5$	297.0759
43	*α*-Glucosylnaringin	$C_{27}H_{32}O_{14}$	579.1704
44	BiochaninA	$C_{16}H_{12}O_5$	283.0603
45	Apigenin	$C_{15}H_{10}O_5$	269.0447
46	Astragalin	$C_{21}H_{20}O_{11}$	447.0921
47	Astraisoflavanin	$C_{23}H_{28}O_{10}$	463.1596
48	Calycosin-7-O-glucoside	$C_{22}H_{22}O_{10}$	445.1128
49	Luteolin-7-O-[(6''-caffeoyl)-*β*-D-glucopyranosyl-(1→6)]-*β*-D-glucopyranoside	$C_{36}H_{36}O_{19}$	771.1761
50	Luteolin-7-O-D-gentibioside	$C_{37}H_{38}O_{18}$	769.1968

Table S2 The compound information used in establishing the PMDF window for saponins in SFI

No.	Identification	Formula	*m/z* [M-H]- (Da)
1	Agroastragaloside Ⅰ	$C_{45}H_{74}O_{16}$	870.0595
2	Agroastragaloside Ⅱ	$C_{43}H_{72}O_{15}$	828.0228
3	Cyclocanthoside E	$C_{41}H_{70}O_{14}$	785.9861
4	Astragaloside Ⅰ	$C_{45}H_{72}O_{16}$	868.0436
5	Astragaloside Ⅱ	$C_{43}H_{70}O_{15}$	826.0069
6	Astragaloside Ⅲ	$C_{41}H_{68}O_{14}$	783.9702
7	Astragaloside Ⅳ	$C_{41}H_{68}O_{14}$	783.9702
8	Astragaloside Ⅴ	$C_{47}H_{78}O_{16}$	898.1126
9	Astragaloside Ⅵ	$C_{47}H_{78}O_{16}$	898.1126
10	Astragaloside Ⅶ	$C_{47}H_{78}O_{16}$	898.1126
11	Astragaloside A	$C_{41}H_{68}O_{14}$	783.9702
12	Astramembranin 2	$C_{35}H_{58}O_{9}$	621.8296
13	Cyclosieversigenin	$C_{30}H_{50}O_{5}$	489.7150
14	Agroastragaloside Ⅲ	$C_{51}H_{82}O_{21}$	1030.1842
15	Agroastragaloside Ⅳ	$C_{49}H_{80}O_{20}$	988.1475
16	Brachyoside B	$C_{36}H_{60}O_{10}$	651.8556
17	Cycloastragenol	$C_{30}H_{50}O_{5}$	489.7150
18	Astrasieversianin 8	$C_{43}H_{70}O_{15}$	826.0069
19	Astramembranoside A	$C_{42}H_{70}O_{15}$	813.9962
20	Isoastragaloside 1	$C_{45}H_{72}O_{16}$	868.0436
21	Isoastragaloside 2	$C_{43}H_{70}O_{15}$	826.0069
22	Isoastragaloside 4	$C_{41}H_{68}O_{14}$	783.9702
23	Huangqiyenin D	$C_{38}H_{62}O_{11}$	693.8923
24	Malonylastragaloside 1	$C_{48}H_{74}O_{19}$	954.0898
25	Astragaloside Ⅷ	$C_{47}H_{76}O_{17}$	912.0961
26	Azukisaponin Ⅴ	$C_{48}H_{78}O_{18}$	942.1221
27	Soyasaponin 1	$C_{48}H_{78}O_{18}$	942.1221
28	Soyasaponin 2	$C_{47}H_{76}O_{17}$	912.0961
29	Cyclounifolioside C	$C_{36}H_{62}O_{10}$	653.8715
30	Astramembranoside B	$C_{41}H_{70}O_{14}$	785.9861
31	Cyclocephaloside	$C_{41}H_{68}O_{14}$	783.9702
32	Huangqiyenin A	$C_{36}H_{58}O_{10}$	649.8397
33	Huangqiyenin B	$C_{36}H_{60}O_{10}$	651.8556
34	Huangqiyenin E	$C_{42}H_{66}O_{14}$	793.9650
35	Huangqiyenin F	$C_{40}H_{64}O_{12}$	735.9290
36	Mongholicoside 1	$C_{36}H_{62}O_{11}$	669.8709
37	Mongholicoside 2	$C_{36}H_{62}O_{11}$	669.8709
38	Methylnissolin	$C_{17}H_{16}O_{5}$	299.3059
39	Licoagroside D	$C_{22}H_{24}O_{10}$	447.4200

40	Daucosterol	$C_{35}H_{60}O_6$	575.8473
41	*β*-sitosterol	$C_{29}H_{50}O$	413.7067
42	Sitosterol	$C_{29}H_{50}O$	413.7067
43	Stigmast5en3ol	$C_{29}H_{50}O$	413.7067
44	Lariciresinol	$C_{20}H_{24}O_6$	359.4010
45	Lupenone	$C_{30}H_{48}O$	423.7015
46	Lupeol	$C_{30}H_{50}O$	425.7174
47	Medicarpin	$C_{16}H_{14}O_4$	269.2800
48	*(3β,6α,16β,20R,24S)*-3-[(3,4-di-O-acetyl-*β*-Dxylopyranosyl)oxy]-20,24-epoxy-16,25-dihydroxy-9,19-cyclolanostan-6-y	$C_{45}H_{71}O_{16}$	866.4748
49	Astraisoflavan-7-O-*β*-D-glucoside	$C_{23}H_{27}O_{10}$	462.1604
50	Odoratin-7-O-*β*-D-glucoside	$C_{23}H_{23}O_{11}$	474.1251
51	Astraisoflavan-7-O-*β*-D-glucosid	$C_{23}H_{27}O_{10}$	462.1604
52	5'-hydroxyisomucronulatol-2',5'-di-O-glucoside	$C_{29}H_{37}O_{16}$	640.2093
53	Complanatuside	$C_{28}H_{31}O_{16}$	622.1623
54	Astraisoflavan-7-O-*β*-D-glucoside	$C_{23}H_{29}O_{10}$	464.1761
55	Isomer of Complanatuside	$C_{28}H_{31}O_{16}$	622.1623
56	Isomer of Astraisoflavan-7-O-*β*-D-glucoside	$C_{23}H_{27}O_{10}$	462.1604
57	Kaempferol-4'-methylether-3-D-glucoside	$C_{22}H_{21}O_{11}$	460.1095
58	Isomer of Kaempferol-4'-methylether-3-D-glucoside	$C_{22}H_{21}O_{11}$	460.1095
59	Isomer of Soyasaponin 1	$C_{48}H_{78}O_{18}$	942.1221
60	Isomer of Astragaloside Ⅱ	$C_{43}H_{70}O_{15}$	826.0069
61	Mongholicoside II	$C_{38}H_{63}O_{11}$	694.4370
62	Isomer of Mongholicoside II	$C_{38}H_{63}O_{11}$	694.4370
63	Azukisaponin V methyl ester	$C_{49}H_{81}O_{18}$	956.5423
64	Cyclocanthoside E	$C_{41}H_{69}O_{14}$	784.4698
65	Ismoer of Cyclocanthoside E	$C_{41}H_{69}O_{14}$	784.4698
66	Isomer of Mongholicoside 2	$C_{47}H_{76}O_{17}$	912.0961
67	Isomer of Mongholicoside 1	$C_{48}H_{78}O_{18}$	942.1221
68	Isomer of Agroastragaloside Ⅳ	$C_{41}H_{68}O_{14}$	783.9702
69	Isomer of Isoastragaloside 1	$C_{45}H_{72}O_{16}$	868.0436
70	Isomer of Huangqiyenin E	$C_{42}H_{66}O_{14}$	793.9650
71	Isomer of Huangqiyenin A	$C_{36}H_{58}O_{10}$	649.8397
72	Isomer of Cyclocanthoside E	$C_{41}H_{70}O_{14}$	785.9861
73	Isomer of Licoagroside D	$C_{22}H_{24}O_{10}$	447.4200
74	Isomer of Brachyoside B	$C_{36}H_{60}O_{10}$	651.8556
75	Isomer of Huangqiyenin D	$C_{38}H_{62}O_{11}$	693.8923
76	Isomer of Isoastragaloside 4	$C_{41}H_{68}O_{14}$	783.9702
77	Isomer of Agroastragaloside Ⅲ	$C_{51}H_{82}O_{21}$	1030.1842
78	Isomer of Cyclosieversigenin	$C_{30}H_{50}O_5$	489.7150
79	Isomer of Astramembranin 2	$C_{35}H_{58}O_9$	620.8296

Table S3 The compound information used in establishing the PMDF window for alkaloids in SFI

No.	Identification	Formula	*m/z* [M-H]$^-$ (Da)
1	Codonopyrrolidium A	C19H28NO4	335.2095
2	Codonopyrrolidium B	C14H22NO4	269.1626
3	Codonopsine	C14H19NO4	266.1391
4	Codonopsinine	C13H17NO3	236.1286
5	Codotubulosine A	C18H26NO6	353.1837
6	Codotubulosine B	C14H22NO4	269.1626
7	Adenosine	C10H13N5O4	268.1045
8	Hypoxanthine	C5H4N4O	137.0463
9	6-Methoxy-quinoline-4-carbaldehyde	C11H9NO2	188.0711
10	Perlolyrine	C16H12N2O2	265.0976
11	N-9-formyl Harman	C45H35N9O6	798.2786
12	1-Carbomethylcarboline	C45H35N9O7	814.2736
13	Norharman	C43H33N9O5	756.2681
14	Nicotine	C10H14N2	163.1234
15	Uracil	C4H4N2O2	113.0351
16	6-Methoxy-4-formyl quinolone	C11H9NO2	188.0711
17	1, 2, 3,4-Tetrahydro-*β*-carboline-3-carboxylic acid	C12H14N2O2	219.1133
18	Tryptophan	C11H12N2O2	205.0976
19	Astragaline A	C10H11NO4	210.0766
20	Astragaline D	C12H15NO5	254.1028
21	Astrafaline E	C14H16N2O5	293.1137
22	Astrafaline F	C12H18N2O5	271.1293
23	Hypaphorine	C14H18N2O2	247.1445
24	1-(1H-Pyrrol-2-yl) ethenone	C6H7NO	110.0605
25	HDTIC-1	C12H15NO5	254.1028
26	HDTIC-2	C12H15NO5	240.0997
27	Uridine	C9H12N2O6	245.0773
28	Adenosine	C10H13N5O4	268.1045
29	Codonopiloside A	C19H29O9N	416.1908
30	Codonopyrrolidium A	C19H27O5N	350.1952

Table S4 The compound information used in establishing the PMDF window for polyene and polytylene components in SFI

No.	Identification	Formula	*m/z* [M-H]⁻ (Da)
1	Lobetyol	C14H18O3	233.1177
2	Lobetyolin	C20H29O9	412.1732
3	Lobetyolinin	C26H38O13	557.2232
4	Pilosulyne A	C14H20O4	251.1282
5	Pilosulyne B	C14H18O4	249.1126
6	Pilosulyne C	C14H20O3	235.1333
7	Pilosulyne D	C20H28O8	395.1705
8	Pilosulyne E	C26H28O13	547.1450
9	Pilosulyne F	C14H20O4	251.1282
10	Cordifolioidyne A	C16H20O6	307.1181
11	Cordifolioidyne B	C16H20O4	275.1282
12	Cordifolioidyne C	C20H28O8	395.1705
13	Codonopilodiynoside A	C20H28O8	395.1705
14	Codonopilodiynoside C	C26H40O15	591.2287
15	Codonopilodiynoside B	C26H40O15	591.2287
16	Codonopilodiynoside D	C20H30O8	397.1861
17	Codonopilodiynoside E	C26H40O12	543.2440
18	Codonopilodiynoside F	C26H38O12	541.2283
19	Codonopilodiynoside G	C26H38O12	541.2283
20	Codonopilodiynoside H	C20H27O8	394.1626
21	Pratialin B	C32H48O18	719.2760
22	Codonopilodiynoside L	C20H28O9	411.1654
23	Codonopilodiynoside M	C20H28O9	411.1654
24	Codonopiloenynenoside A	C20H30O9	413.1810
25	Tetradeca-*(4E,8E,12E)*-triene-10-yne-1,6,7-triol	C14H20O3	235.1333
26	Codonopiloenynenoside B	C22H36O10	459.2228
27	(+)-*(6R,7R,12E)*-Tetradeca-12-en-10-yne-1,6,7-triol	C14H24O3	239.1646
28	*(2E,6E)*-Octa-2,6-dien-4-ynoic acid	C8H8O2	135.0446
29	*(E)*-Oct-6-en-4-ynoic acid	C8H10O2	137.0602
30	(+)-*(2R,7S)*-1,7-Dihyrdroxy-2,7-cyclotetradeca-4,8,12-trien-10-yn-6-one	C14H16O3	231.1020
31	9-(tetrahydropyran-2-yl)-non-trans-2,8-diene-4,6-diyn-1-ol	C14H16O2	215.1071

32	9-(tetrahydropyran-2-yl)-non-trans-8-ene-4,6-diyn-1-ol	$C_{14}H_{18}O_2$	217.1228
33	Pilosulinene A	$C_{14}H_{12}O_2$	211.0758
34	Pilosulinol A	$C_{22}H_{28}O_7$	403.1755
35	Pilosulinol B	$C_{22}H_{28}O_7$	403.1755
36	Tangshenyne A	$C_{20}H_{28}O_{10}$	427.1603
37	Choushenpilosulyne	$C_{30}H_{48}O_4$	471.3472
38	Choushenpilosulyne A	$C_{37}H_{58}O_9$	645.4000
39	Choushenpilosulyne B	$C_{37}H_{58}O_9$	645.4000

Table S5 Detailed information about the characterized compounds in SFI

NO.	Type	tR (min)	Identification	Formula	RDB	Error (ppm)	$[M+H]^+$	$[M+Na]^+$	$[M-H]^-$	$[M+CHOO]^-$	Major fragment ions	CCS (angstrom^2)	Source
1		3.66	Complanatuside	C28H32O16	12.5	-2.02			623.1606	669.1661	$[M-H]^-$: 461.11, 299.06, 151.04	230.06	As, Co
2		5.62	Kaempferol-3-O-sophoroside	C27H30O16	12.5	-1.72			609.1450		$[M-H]^-$: 447.09, 327.05, 285.04, 193.05	227.92	As, Co
3		5.72	Astragaloside	C28H32O17	13.5	0.67			639.1571	685.1616	$[M-H]^-$: 579.18, 477.13, 447.11, 315.05	236.72	As, SF1
4		7.81	Unknown	C37H52O20	12.5	-1.00			815.2966	861.3030	$[M-H]^-$: 653.24, 521.20, 461.11	258.30	As, SF1
5		8.68	Emodin-di-Glucoside	C27H30O15	13.5	-2.58			593.1497		$[M-H]^-$: 575.14, 503.12, 473.11, 383.08, 353.07	246.21	As, SF1
**6**		**10.50**	**Pratensein-7-O-*β*-D-glucopyranoside**	**C22H22O11**	**12.5**	**-1.55**			**461.1108**		**$[M-H]^-$: 299.06, 284.03, 266.02**	**204.58**	As, Co, SF1
7		10.88	Prunin	C21H22O10	11.5	-1.06			433.1136		$[M-H]^-$: 271.06, 256.02, 193.05, 135.00	177.80	As, Co
8		11.54	Unknown	C33H44O17	12.5	-1.24			711.2497	757.2550	$[M-H]^-$: 531.19, 369.13, 263.24, 243.15	245.29	As, SF1
9		11.68	Tangshenoside I	C29H42O18	9.5	-1.68			677.2287		$[M-H]^-$: 523.23, 497.16, 453.18, 261.10	236.52	Co
10	Flavonoids	13.05	Unknown	C31H42O15	11.5	-1.92			653.2438	699.2493	$[M-H]^-$: 491.19, 329.14, 315.12, 311.13	232.10	As, SF1
11		13.72	Unknown	C28H32O15	13.5	0.85			607.1668	653.1725	$[M-H]^-$: 329.15, 283.06, 268.04	232.10	As, SF1
12		13.78	Astragalin	C22H24O10	11.5	-0.15			447.0879		$[M-H]^-$: 285.04, 255.03, 193.05	198.00	As, Co, SF1
13		13.96	Isomucronulatol-2′,5′-di-O-glucoside	C28H34O16	12.5	-0.89			641.1718		$[M-H]^-$: 479.12, 317.07, 251.03, 191.03	250.35	As, Co, SF1
**14**		**14.78**	**Calycosin-7-O-*β*-D-glucopyranoside**	**C22H22O10**	**11.5**	**-2.47**			**445.1130**	**491.1198**	**$[M-H]^-$: 283.07, 268.08, 207.05**	**224.57**	**As, SF1**
15		15.08	Unknown	C31H42O15	11.5	-1.46			653.2441	699.2493	$[M-H]^-$: 491.19, 329.14, 315.12, 311.13	225.32	As, SF1
16		15.89	Kumatakenin	C17H14O7	11.5	-0.93			313.0715		$[M-H]^-$: 298.05, 283.02, 255.03, 233.06, 193.05	172.98	SF1
17		16.20	Kumatakenin+2Glc	C29H34O16	13.5	-2.27			637.1760	683.2500	$[M-H]^-$: 475.12, 313.07, 255.07	227.70	As, SF1
18		16.57	6′-O-Acetycalycosin-7-O-*β*-D-glucoside+Glc	C28H42O17	8.5	1.69			649.2355	695.2407	$[M-H]^-$: 487.18, 469.17, 353.11, 293.09, 179.06	259.40	As, SF1
19		17.04	Neohesperidin	C28H36O15	11.5	-1.23			611.1974		$[M-H]^-$: 449.15, 287.09, 272.06, 255.07, 165.06,	227.90	As, SF1

								135.05, 121.03		
20	17.66	Apigenin 7-glucoside	C21H20O10	12.5	-1.16	431.0979	477.1033	$[M-H]^-$: 269.05, 225.10, 199.40	207.25	As, Co
21	19.30	Lobetyolinin	C26H38O13	8.5	0.06	557.2240	603.2294	$[M-H]^-$: 539.21, 467.18, 323.10	246.12	Co.SF1
22	19.31	8,2'-Dihydroxy-7,4'-dimethoxyisoflavan	C17H18O6	9.5	-2.72	317.1022		$[M-H]^-$: 267.08, 241.05, 193.05, 137.02, 121.03	170.95	As, SF1
23	20.38	Unknown	C30H36O17	13.5	-0.44	667.1877		$[M-H]^-$: 505.20, 417.18, 402.17, 329.14, 166.03	238.79	As, SF1
24	21.46	Sissotrin	C22H22O10	12.5	-3.63	445.1133		$[M-H]^-$: 283.06, 253.05, 239.04	200.28	As, SF1
25	22.03	Unknown	C29H38O15	11.5	1.29	625.2146	671.2202	$[M-H]^-$: 463.16, 301.11, 286.08, 269.05, 146.06	230.05	**As, SF1**
26	22.22	Naringenin7Rhamnoglucoside	C27H32O14	12.5	1.59	579.1723	625.2141	$[M-H]^-$: 459.11, 313.07, 271.06, 235.02, 151.00	212.37	**As, Co**
27	22.79	Odoratin-7-O-Glc	C23H26O11	11.5	-1.10	477.1397		$[M-H]^-$: 401.14, 386.13, 355.04, 178.03	213.42	As, SF1
28	23.17	6-Methoxyflavanone	C16H14O3	10.5	-1.80	285.0758		$[M-H]^-$: 267.09, 233.06, 193.05, 149.06	158.22	As, SF1
29	23.88	Isomer of 33	C28H34O15	12.5	-2.21	609.1812		$[M-H]^-$: 301.07, 286.05, 257.08, 242.06	239.25	As, Co
30	23.91	6′-O-Acetycalycosin-7-O-*β*-D-glucoside	C24H24O11	13.5	1.22	487.1252	533.1310	$[M-H]^-$: 325.07, 283.06, 268.04, 239.04	219.30	As, SF1
31	25.87	Isomer of 30	C28H34O15	12.5	-1.11	609.1818		$[M-H]^-$: 447.13, 301.11, 286.05, 257.08	223.40	As, Co
**32**	**28.85**	**Ononin**	**C22H22O9**	**11.5**	**-1.46**	**429.1187**	**475.1244**	**$[M-H]^-$: 269.08, 253.05, 225.05**	**220.24**	**As, Co**
33	29.21	6′-O-Acetycalycosin-7-O-*β*-D-glucoside	C24H24O11	13.5	-0.71	487.1249	533.1309	$[M-H]^-$: 325.07, 297.08, 283.06, 268.01	224.62	**As, Co**
34	30.22	Afrormosin	C17H14O5	11.5	-2.18	297.0762		$[M-H]^-$: 267.06, 233.06, 193.05	164.66	As, SF1
35	30.53	Trihydroxy-dimethoxy dihydroisoflavone	C22H24O12	11.5	-0.11	479.1189		$[M-H]^-$: 461.13, 317.13, 273.05, 255.05	206.62	As, SF1
36	32.27	Calycosin derivative	C28H34O14	12.5	-0.23	593.1874	639.1928	$[M-H]^-$: 299.06, 284.07, 269.05	266.34	As, SF1
37	32.51	3-O-Methylorobol/Calycosin	C16H12O6	11.5	3.30	299.0560		$[M-H]^-$: 284.05, 256.03, 227.03, 135.04	166.86	As, SF1
38	32.63	287.09+Glu	C22H26O10	10.5	-1.20	449.1448	495.1656	$[M-H]^-$: 287.09, 272.07, 255.07, 165.06, 135.04, 121.03, 109.03	202.48	As, SF1
39	32.65	Isomucronulatol derivative	C16H16O5	9.5	-4.87	287.0932		$[M-H]^-$: 267.06, 223.06, 149.02	162.66	As, SF1
**40**	**33.55**	**9,10-Dimethoxypterocarpan-3-O-*β*-D-glucoside**	**C23H26O10**	**10.5**	**-1.43**	**461.1453**	**507.1510**	**$[M-H]^-$: 301.11, 271.06, 197.06, 167.07**	**228.92**	**As, SF1**
41	33.69	Astrapterocarpan-7-O-*β*-D-glucopyranoside	C23H26O10	11.5	-1.26	461.1447	507.1500	$[M-H]^-$: 461.14, 299.09, 284.07, 235.24	228.92	As, SF1
42	34.10	Vesticarpan derivative	C17H16O7	9.5	-0.94	331.1184		$[M-H]^-$: 301.11, 267.06, 223.06, 193.05	177.40	As, SF1
43	34.19	301+Glc+Api	C28H36O14	11.5	-1.09	595.2026		$[M-H]^-$: 463.16, 301.11, 286.08, 271.06, 179.07	228.03	As, SF1

**44**		**36.10**	**Calycosin**	**C16H12O5**	**11.5**	**1.60**	**283.0616**		**$[M-H]^-$: 268.05, 239.04, 211.04, 195.05, 183.05, 135.01**	**162.76**	**As, Co, SF1**
45		36.20	Unknown	C24H30O11	10.5	-0.48	493.1713		$[M-H]^-$: 463.16, 301.11, 286.08, 147.05	213.25	As, Co
46		36.67	7,2'-Dihydroxy-3', 4'-dimethoxyisoflavan	C17H18O5	9.5	-1.70	301.1071		$[M-H]^-$: 286.08, 223.06, 193.05, 179.07, 149.02, 121.03	166.81	As.Co.SF1
**47**		**36.72**	**Isomucronulatol-7-O-glucoside**	**C23H28O10**	**10.5**	**0.35**	**463.1611**	**509.1663**	**$[M-H]^-$: 301.11, 286.09, 271.06, 147.04**	**215.86**	**As, Co, SF1**
48		36.77	Neocomplanoside+2H	C25H30O11	11.5	2.60	505.1723	551.1780	$[M-H]^-$: 445.15, 301.11, 286.08, 135.05	214.88	As, Co, SF1
49		38.80	Unknown	C28H34O15	12.5	-2.21	609.1812		$[M-H]^-$: 301.07, 286.05, 257.08, 242.06	223.41	As, Co
50		41.27	9,10-Dimethoxypterocarpan-3-O-malonyl-Glc	C25H28O11	12.5	2.49	503.1566	549.1623	$[M-H]^-$: 486.30, 299.09, 284.07, 269.05	217.63	As, SF1
51		43.54	9,10-Dimethoxypterocarpan-3-O-malonyl-Glc+2H	C25H30O11	11.5	1.16	505.1724	551.1771	$[M-H]^-$: 445.15, 343.12, 301.11, 286.08, 179.07	224.41	As, Co, SF1
52		44.35	Unknown	C30H36O15	13.5	-2.45	635.1966		$[M-H]^-$: 593.19, 575.18, 299.09, 284.07, 269.05	254.95	As, SF1
53		44.73	Dihydroxy-dimethoxy dihydroisoflavone	C17H16O6	10.5	1.32	315.0873		$[M-H]^-$: 215.13, 195.05	176.62	As, SF1
**54**		44.77	Formononetin-7-O-Glc-6''-O-acetate derivative	C25H26O12	13.5	-0.39	517.1350		$[M-H]^-$: 267.07, 252.04, 223.04, 195.05	214.78	As, SF1
55		44.82	6″-O-acetyl-ononin	C24H24O10	13.5	-0.05	471.1291	517.1352	$[M-H]^-$: 449.28, 327.22, 315.09, 267.07	218.02	As
56		46.71	Astraisoflavan-malonyl-Glc	C26H30O13	12.5	1.05	549.1613		$[M-H]^-$: 299.06, 284.05, 267.06	217.04	As, SF1
57		48.53	Isomer of 9,10-Dimethoxypterocarpan-3-O-malonyl-Glc+2H	C25H30O11	11.5	-1.87	505.1706	551.1759	$[M-H]^-$: 445.15, 343.12, 301.11, 286.08	222.14	As, Co, SF1
58		51.54	Formononetin	C16H12O4	11.5	4.70	267.0664		$[M-H]^-$: 252.04, 233.05, 195.06, 132.03	156.45	As, Co, SF1
59		22.97	Unknown	C54H93O28	8.5	0.67	1189.5867		$[M-H]^-$: 1019.34, 711.25, 521.67, 493.14, 401.15, 270.05, 197.81	325.49	As, Co, SF1
60	Saponins	24.55	Unknown	C41H70O15	7.5	-1.17	801.4633	847.4667	$[M-H]^-$: 639.41, 507.37, 489.36	272.93	As, SF1
61		29.62	Unknown	C41H70O15	7.5	-2.93	801.4619	847.4667	$[M-H]^-$: 639.41, 507.37, 489.36	274.34	As, SF1

62	30.48	Unknown	C53H92O25	8.5	-1.12	1127.5836	1173.5896	$[M-H]^-$: 965.53, 819.47, 687.43	321.08	As, SF1
63	30.62	Unknown	C42H74O16	6.5	-1.09	833.4895	879.4949	$[M-H]^-$: 671.40, 509.39, 469.35, 425.30	278.73	As, SF1
64	37.72	Bicusposide F	C37H62O11	7.5	1.43	681.4229	727.4281	$[M-H]^-$: 621.40, 603.38, 329.23	277.44	As, SF1
65	38.84	Unknown	C42H70O17	8.5	1.11	845.4544	891.3503	$[M-H]^-$: 799.45, 637.21, 161.05, 119.03	278.67	As, SF1
66	40.02	Unknown	C54H88O24	11.5	-0.39	1119.5588		$[M-H]^-$: 10101.55, 1057.56, 911.50, 587.39, 473.37	344.23	As, SF1
67	41.48	Unknown	C42H70O15	8.5	3.13	813.4667	859.4700	$[M-H]^-$: 771.32, 577.24, 389.17, 341.18	285.46	As, SF1
68	42.78	Unknown	C53H88O24	10.5	-1.28	1107.5579	1153.5638	$[M-H]^-$: 945.50, 783.45, 621.40, 489.36	314.24	As, Co, SF1
69	43.04	Unknown	C47H82O18	7.5	-0.45	933.5424	979.5471	$[M-H]^-$: 787.48, 655.44, 493.39	294.26	As, SF1
70	43.07	Unknown	C47H76O19	10.5	-0.41	943.4904	989.4962	$[M-H]^-$: 781.44, 619.39, 487.35	294.22	As, Co
**71**	**43.55**	**Astragaloside VII**	**C47H78O19**	**9.5**	**3.40**	**945.5091**	**991.5159**	**$[M-H]^-$: 783.45, 621.40, 489.36**	**294.21**	**As, SF1**
72	44.53	Robinioside E	C48H78O19	10.5	1.23	957.5076		$[M-H]^-$: 939.55, 895.57, 811.50, 649.55, 631.40, 473.42, 453.37	321.77	As, SF1
73	44.74	Unknown	C48H80O20	9.5	-3.74	975.5134	1021.5200	$[M-H]^-$: 813.46, 651.41, 489.36	314.57	As, SF1
**74**	**45.16**	**Astragaloside VI**	**C47H78O19**	**9.5**	**-1.40**	**945.5051**	**991.5104**	**$[M-H]^-$: 783.45, 621.40, 489.36, 471.35, 383.32**	**317.21**	**As, SF1**
75	45.91	Astragaloside V/VI/VII+C2H2	C48H76O20	11.5	1.51	971.4872	1017.4926	$[M-H]^-$: 909.58, 763.52, 645.45, 469.40	324.02	As, Co, SF1
76	46.08	Unknown	C47H74O19	11.5	-1.68	941.4736		$[M-H]^-$: 923.46, 879.48, 733.42, 601.37, 439.33	317.23	As, SF1
77	46.26	Unknown	C47H80O19	8.5	-2.39	947.5198	993.5301	$[M-H]^-$: 785.47, 623.41, 491.37, 473.37	330.85	As, SF1
78	46.37	Unknown	C48H76O20	11.5	0.13	971.4858	1017.4916	$[M-H]^-$: 909.58, 763.52, 645.44, 469.38	324.02	As, Co, SF1
79	46.85	Cyclocanthoside E	C41H70O14	7.5	-0.52	785.4689	831.4752	$[M-H]^-$: 623.42, 491.37, 473.33, 415.32, 397.31, 323.28	267.58	As, SF1
80	47.25	Unknown	C46H76O18	9.5	-1.98	915.4941	961.5018	$[M-H]^-$: 829.46, 783.50, 597.46, 403.31	298.73	As, SF1

81	47.29	3-O-rhap-galp-glup-conplogenin	C48H76O19	11.5	-1.55	955.4905	1001.4964	[M-H]⁻: 937.43, 893.47, 809.44, 747.42, 629.35, 539.34, 471.33	321.78	As, SF1
82	47.49	Astrasieversianin XIV	C47H78O18	9.5	1.76	929.5126	975.5184	[M-H]⁻: 829.46, 809.31, 612.30, 480.18	294.28	As, SF1
83	48.07	Lancemasides D	C52H79O24	13.5	-0.99	1087.4956		[M-H]⁻: 977.53, 911.44, 831.47, 677.03, 197.81	324.29	As, SF1
84	48.12	Astrasieversianin XIV+2H	C47H80O18	8.5	-2.06	931.5253	977.5309	[M-H]⁻: 785.47, 769.47, 623.42, 607.42, 491.38	298.86	As, SF1
85	48.30	Isomer of Pardarinoside B	C42H70O15	8.5	-1.60	813.4628	859.4689	[M-H]⁻: 651.41, 489.36, 605.31, 327.22	283.17	As, SF1
86	48.31	Pardarinoside B	C42H70O15	8.5	-3.68	813.4612	859.4706	[M-H]⁻: 667.41, 327.22, 309.21	283.17	As, SF1
87	48.33	Sieberoside	C42H70O15	8.5	-2.96	813.4618	859.4679	[M-H]⁻: 651.41, 489.36, 327.22, 211.11	283.17	As, SF1
88	48.81	Unknown	C41H70O14	7.5	-0.45	785.4689	831.4744	[M-H]⁻: 623.42, 491.37, 311.31	301.69	As, SF1
89	48.83	Astragaloside V/VI/VII+C3H6	C49H80O20	10.5	-1.04	987.5160	1033.5222	[M-H]⁻: 927.50, 765.44, 633.40, 603.39, 489.36	317.01	As, SF1
90	49.21	Unknown	C66H69O8	32.5	0.21	989.5000		[M-H]⁻: 827.41, 809.43, 393.20, 327.22, 197.81	333.56	As, SF1
91	49.26	Unknown	C47H76O19	10.5	-0.86	943.4901	989.4952	[M-H]⁻: 781.44, 619.38, 487.35	310.1	As, SF1
**92**	**49.90**	**Astragaloside V**	**C47H78O19**	**9.5**	**1.18**	**945.5052**	**991.5140**	**[M-H]⁻: 783.45, 621.40, 489.36, 471.35, 383.32**	**328.55**	**As, SF1**
93	50.88	Unknown	C48H74O20	12.5	-0.98	969.4691	1015.4752	[M-H]⁻: 907.47, 761.41, 599.36, 437.30	321.72	As, SF1
94	50.88	Unknown	C49H84O20	8.5	-0.29	991.5469	1037.5536	[M-H]⁻: 931.53, 785.47, 653.43	323.53	As, SF1
95	50.92	Agroastragaloside II	C43H72O15	8.5	4.41	827.4835	873.4855	[M-H]⁻: 767.51, 635.43, 605.39, 443.27	292.51	As, SF1
96	51.25	Unknown	C47H72O19	12.5	-1.65	939.4579		[M-H]⁻: 877.46, 731.40, 643.35, 599.39, 485.33	314.93	As, SF1
97	51.38	Unknown	C42H68O16	9.5	0.03	781.4368	827.4435	[M-H]⁻: 649.43, 619.40, 487.38	260.77	As, SF1
**98**	**51.59**	**Astragaloside IV**	**C41H68O14**	**8.5**	**-1.30**	**783.4528**	**829.4576**	**[M-H]⁻: 621.40, 489.36, 471.35, 415.29, 383.29, 276.62**	**297.1**	**As, SF1**
99	51.91	Unknown	C49H80O20	10.5	-3.14	987.5119	1033.5226	[M-H]⁻: 927.50, 765.44, 633.40, 603.34, 489.35	333	As, SF1
**100**	**51.70**	**Astragaloside III**	**C41H68O14**	**8.5**	**2.90**	**783.4559**	**829.4604**	**[M-H]⁻: 769.61, 685.51, 651.36, 503.25**	**297.1**	**As, SF1**
**101**	**52.08**	**Isoastragaloside IV**	**C41H68O14**	**8.5**	**4.26**	**783.4564**	**829.4608**	**[M-H]⁻: 621.40, 489.36, 383.28**	**297.1**	**As, SF1**
102	52.22	Unknown	C41H70O13	7.5	-1.11	769.4735	815.4795	[M-H]⁻: 769.48, 607.42, 475.38, 399.32	290.31	As, Co
103	52.46	Unknown	C51H82O21	11.5	2.02	1029.529	1075.5348	[M-H]⁻: 969.51, 909.49, 747.46	328.19	As, SF1

						7				
104	52.61	Unknown	C41H68O13	8.5	-2.04	767.4572	813.4625	$[M-H]^{-}$: 637.34, 329.23, 311.21, 201.08	267.69	As, SF1
**105**	**52.95**	**Rajanoside**	**C43H70O15**	**9.5**	**-0.32**	**825.4639**	**871.4705**	**$[M-H]^{-}$: 783.46, 765.44, 717.42, 603.41, 489.36**	**285.64**	**As, SF1**
**106**	**53.09**	**Soyasaponin I**	**C48H78O18**	**10.5**	**-1.88**	**941.5098**	**987.5147**	**$[M-H]^{-}$: 923.51, 795.46, 733.45, 615.40, 597.39, 457.37, 437.36**	**335.51**	**As, Co, SF1**
107	53.39	Unknown	C43H68O15	10.5	-1.63	823.4472	869.4538	$[M-H]^{-}$: 763.43, 601.37, 469.39	306.08	As, SF1
108	53.59	Astragaloside II	C44H72O17	9.5	1.33	871.4703	917.5026	$[M-H]^{-}$: 825.48, 765.45, 633.36, 445.06	306.07	As, SF1
109	53.66	Cycloaraloside A	C36H60O10	7.5	-3.94	651.4093	697.4177	$[M-H]^{-}$: 489.40, 487.26, 329.23, 171.10	259.03	As, Co, SF1
110	53.66	Unknown	C47H70O16	13.5	4.37	889.4630	935.4986	$[M-H]^{-}$: 869.46, 691.35, 475.29, 329.23	319.76	As, Co, SF1
111	53.88	Unknown	C48H76O19	11.5	1.39	955.4921	1001.4992	$[M-H]^{-}$: 937.48, 893.49, 633.40	324.09	As, Co, SF1
112	53.95	Agroastragaloside IV	C49H80O20	10.5	0.81	987.5173	1032.5471	$[M-H]^{-}$: 941.51, 715.49, 615.43, 457.41	335.51	As, Co, SF1
113	54.01	Foetidissimoside A	C52H81O22	12.5	0.72	1057.521 9		$[M-H]^{-}$: 1011.52, 941.51, 871.47, 665.39, 485.24, 257.13	323.26	As
**114**	**54.48**	**Isoastragaloside II**	**C43H70O15**	**9.5**	**2.49**	**825.4657**	**871.4694**	**$[M-H]^{-}$: 783.46, 621.40, 603.39, 489.35**	**310.67**	**As, SF1**
115	54.54	Unknown	C41H66O13	9.5	2.54	765.4445	811.4407	$[M-H]^{-}$: 719.28, 629.19, 485.11, 383.09	286	As, SF1
116	54.82	Unknown	C48H78O17	10.5	-1.15	925.5156	971.5206	$[M-H]^{-}$: 907.50, 863.51, 717.46, 537.39, 509.40, 439.36	317.31	As, SF1
**117**	**55.14**	**Cyclocephaloside Ⅱ**	**C43H70O15**	**9.5**	**2.11**	**825.4654**	**871.4692**	**$[M-H]^{-}$: 783.47, 765.44, 603.39, 489.35**	**294.82**	**As, SF1**
118	55.32	Unknown	C45H74O16	9.5	-0.69	869.4893	915.4948	$[M-H]^{-}$: 767.46, 605.41, 473.44	331.34	As, Co, SF1
119	55.39	Unknown	C48H76O18	11.5	0.02	939.4959	985.4992	$[M-H]^{-}$: 921.47, 877.50, 523.40, 793.46, 731.46, 613.37, 455.33	317.24	As, Co, SF1

120		55.45	Cyclogaleginoside A+C5H8O4	C35H72O19	0.5	3.26			795.4615	841.4589	[M-H]-: 633.31, 615.38, 457.37	301.93	As, SF1
121		55.62	Cyclogaleginoside A	C37H60O10	8.5	2.59			663.4125	709.4170	[M-H]-: 617.32, 435.00, 315.00, 293.21	207.31	As, SF1
122		55.69	Unknown	C54H84O21	13.5	1.01			1067.5443	1113.5503	[M-H]-: 1049.54, 821.53, 641.43, 483.48	339.61	As, Co
123		55.74	Unknown	C47H76O16	10.5	-1.38			895.5048		[M-H]-: 877.49, 749.45, 537.39, 509.40, 439.36	315.16	As, SF1
124		56.18	Unknown	C37H16O18	8.5	2.58			747.0248	793.4385	[M-H]-: 709.42, 629.39, 409.27, 311.22, 293.20	301.92	As, Co, SF1
125		56.57	Unknown	C36H58O9	8.5	0.80			633.4013	679.0258	[M-H]-: 545.30, 487.26, 327.21, 309.21	254.63	As
126		56.60	Unknown	C45H74O15	9.5	-0.58			853.4950	899.4997	[M-H]-: 827.42, 707.39, 647.32, 605.38, 491.34	301.56	As, SF1
**127**		**57.15**	**Isoastragaloside I**	**C45H72O16**	**10.5**	**0.46**			**867.4752**	**913.4812**	**[M-H]-: 765.44, 717.42, 633.40, 489.36**	**326.62**	**As, SF1**
128		57.37	Acetylastragaloside I	C47H74O17	11.5	2.85			909.4874	955.5592	[M-H]-: 867.58, 849.61, 825.60, 807.58, 747.53	377.98	As, SF1
129		57.39	Unknown	C48H74O19	12.5	1.60			953.4767		[M-H]-: 935.44, 909.48, 891.747.43, 489.58	333.36	As, Co
130		1.94	Codonopiloside A	C19H29O9N	5.5	-4.26	416.1908	438.1748			[M+H]+: 398.18, 254.14, 236.13, 218.12, 205.09, 161.06	190.32	Co, SF1
131		1.98	Unknown	C16H23O7N	5.5	-1.52	342.1542	364.1362			[M+H]+: 324.14, 306.13, 250.14, 176.09, 161.06, 146.08	173.32	Co, SF1
132		2.03	Unknown	C15H21O7N	5.5	-2.83	328.1382				[M+H]+: 310.13, 292.12, 282.13, 162.08, 132.07	168.99	Co, SF1
133	Alkaloids	2.24	Unknown	C18H27O9N	5.5	-2.85	402.1747				[M+H]+: 384.16, 240.12, 222.11, 204.10, 146.08, 104.07	186	As, SF1
134		2.36	Unknown	C15H21O7N	5.5	-4.50	328.1382				[M+H]+: 310.13, 292.12, 221.08, 132.07	168.99	As, SF1
135		2.87	Isomer of Codonopiloside A	C19H29O9N	5.5	-4.34	416.1908	438.172			[M+H]+: 398.18, 254.14, 236.13, 218.12, 205.09, 161.06	190.32	Co, SF1
136		3.01	Codonopiloside+CH2	C20H31O9N	5.5	-4.75	430.2062				[M+H]+: 412.19, 268.15, 250.14, 232.13, 205.09, 161.06	182.49	Co, SF1

137	3.41	Isomer of Codonopiloside A	$C_{19}H_{29}O_{9}N$	5.5	-4.94	416.1906	438.1729	$[M+H]^+$: 398.18, 254.14, 236.13, 218.12	199.34	Co, SF1
138	4.50	Unknown	$C_{16}H_{21}O_{7}N$	6.5	-3.61	340.1384		$[M+H]^+$: 325.53, 203.07, 185.04, 115.58	173.31	Co, SF1
139	5.24	Unknown	$C_{25}H_{23}O_{5}N_{2}$	15	-0.15	432.1679	454.1494	$[M+H]^+$: 380.13, 282.10, 268.15, 252.09, 170.11, 159.44	199.11	As, SF1
140	5.27	Unknown	$C_{18}H_{23}O_{8}N$	7.5	-2.44	382.1493		$[M+H]^+$: 364.14, 354.15, 258.10, 186.08, 158.08, 140.07	181.77	Co, SF1
141	6.09	Unknown	$C_{21}H_{37}O_{10}N$	3.5	-2.03	464.2481		$[M+H]^+$: 301.07, 286.11, 258.11, 239.13, 190.12, 165.09, 137.06	205.41	As, Co
142	6.26	Unknown	$C_{29}H_{41}O_{16}N$	9.5	-1.65	660.2493		$[M+H]^+$: 445.15, 427.14, 385.13, 319.12, 177.05, 149.06	241.02	As, SF1
143	6.44	Unknown	$C_{22}H_{37}O_{13}N$	4.5	-1.75	524.2334		$[M+H]^+$: 507.21, 426.21, 345.15, 183.10, 165.09, 144.81	211.41	Co, SF1
144	6.71	Codonopiloside+CH2	$C_{20}H_{31}O_{9}N$	5.5	0.65	430.2069		$[M+H]^+$: 396.20, 287.12, 215.11, 197.10, 171.08, 128.06	210.34	Co, SF1
145	7.33	Unknown	$C_{34}H_{43}O_{13}N$	13.5	-1.28	674.2798	696.2684	$[M+H]^+$: 656.27, 638.26, 478.21, 460.20, 355.12, 387.14, 280.12, 233.08, 161.06	188.01	Co, SF1
146	8.54	Unknown	$C_{15}H_{19}O_{6}N$	6.5	0.59	310.1283		$[M+H]^+$: 292.12, 282.13, 274.11, 264.12, 221.08	167.06	As, Co, SF1
147	8.57	Unknown	$C_{21}H_{35}O_{11}N$	4.5	-2.34	478.2272		$[M+H]^+$: 450.20, 442.22, 373.13, 346.19, 268.10, 235.17, 166.98	209.7	Co, SF1
148	8.71	Unknown	$C_{15}H_{19}O_{6}N$	6.5	-2.78	310.1282		$[M+H]^+$: 292.12, 282.13, 211.08, 161.06, 114.05	167.06	Co, SF1
149	9.06	Unknown	$C_{15}H_{19}O_{6}N$	6.5	-0.59	310.1283		$[M+H]^+$: 292.12, 282.13, 274.11, 264.12, 221.08, 166.12	167.06	Co, SF1

150	12.37	Unknown	C19H29O6N	5.5	-1.83	368.2061	[M+H]+: 350.20, 266.19, 221.08, 184.13, 177.05	186.56	Co, SF1
151	12.81	Unknown	C18H27O5N	5.5	-2.89	338.1958	[M+H]+: 250.14, 205.08, 161.06, 133.05	171.26	Co, SF1
152	13.43	Unknown	C28H37O15N	10.5	2.27	628.2227	[M+H]+: 449.14, 413.12, 287.09, 177.05, 153.05, 147.04	236.9	As, SF1
153	13.44	Unknown	C28H37O15N	10.5	-1.39	628.2227	[M+H]+: 449.14, 319.60, 287.09, 255.06, 177.05, 153.05, 147.04, 123.04	236.9	As, SF1
154	15.81	Unknown	C19H29O6N	5.5	-1.94	368.2061	[M+H]+: 320.19, 266.14, 248.13, 202.14, 172.13	193.37	Co, SF1
155	16.69	Unknown	C28H39O15N	9.5	-3.09	630.2379	[M+H]+: 451.16, 355.12, 289.11, 167.07, 153.05, 144.86	197.19	As, SF1
156	17.32	Codonopyrrolidium A	C19H27O5N	6.5	1.55	350.1952	[M+H]+: 268.15, 250.14, 220.13, 205.09, 161.06	186.7	As, Co, SF1
157	19.23	Unknown	C26H41O13N	6.5	-1.47	578.2642	[M+H]+: 397.18, 217.12, 199.11, 161.06	179.65	As, Co, SF1
158	19.46	Codonopyrrolidium A+2H	C19H29O5N	5.5	-1.84	352.2112	[M+H]+: 334.20, 250.14, 232.13, 205.09, 161.06	186.86	Co, SF1
159	20.40	Unknown	C28H41O11N	8.5	-1.77	568.27	[M+H]+: 466.26, 388.21, 331.15, 313.14, 287.13, 255.10, 189.09, 151.07	235.24	Co, SF1
160	20.46	Isomer of Codonopyrrolidium A+2H	C19H29O5N	5.5	-4.40	352.2103	[M+H]+: 334.20, 250.14, 232.13, 205.09, 161.06	186.86	Co, SF1
161	21.26	Isomer of Codonopyrrolidium A	C19H27O5N	6.5	2.44	350.1955	[M+H]+: 268.15, 250.14, 205.09, 161.06	186.9	Co, SF1
162	21.66	Unknown	C29H41O15N	9.5	2.68	644.2537	[M+H]+: 429.15, 369.13, 303.12, 167.07, 149.06, 123.04	245.5	As, SF1

163	21.86	Unknown	C24H39O12N	5.5	-1.91	534.2535	$[M+H]^+$: 457.24, 402.21, 340.16, 291.12, 219.10, 195.10, 163.07, 135.08	229	As, SF1
164	23.09	Unknown	C24H31O10N	9.5	-3.42	494.2009	$[M+H]^+$: 449.14, 342.72, 287.09, 177.05, 153.05, 147.04	207.48	As, SF1
165	23.49	Isomer of Codonopyrrolidium A+2H	C19H29O5N	5.5	-2.70	352.2109	$[M+H]^+$: 334.20, 250.14, 232.13, 202.14, 172.13	186.86	Co, SF1
166	24.73	Isomer of Codonopyrrolidium A+2H	C19H29O5N	5.5	-4.97	352.2101	$[M+H]^+$: 334.20, 250.14, 232.13, 202.14, 172.13	186.86	Co, SF1
167	24.83	Isomer of Codonopyrrolidium A+2H	C19H29O5N	5.5	-3.71	352.2111	$[M+H]^+$: 250.14, 202.14, 172.13	186.86	Co, SF1
168	24.96	Unknown	C34H47O19N	11.5	-1.90	774.2806	$[M+H]^+$: 463.16, 457.37, 301.11, 269.08, 167.07, 147.04	276.98	As, SF1
169	25.48	Unknown	C20H31O8N	5.5	-2.57	414.2112	$[M+H]^+$: 397.18, 361.16, 235.13, 217.12, 199.11, 161.10	206.11	Co, SF1
170	31.37	Unknown	C28H37O14N	10.5	-2.27	612.2278	$[M+H]^+$: 463.16, 301.11, 269.08, 167.07, 147.04	179.42	As, SF1
171	31.61	Unknown	C29H39O15N	10.5	-1.50	642.2383	$[M+H]^+$: 625.21, 463.16, 301.11, 269.08, 207.07, 191.07, 167.07, 147.04	225.84	As, SF1
172	33.67	Unknown	C35H27O2N2	23	0.21	508.215	$[M+H]^+$: 480.18, 463.16, 269.08, 167.07, 147.04	213.73	As, SF1
173	36.71	Unknown	C23H31O10N	8.5	-1.04	482.2016	$[M+H]^+$: 465.17, 429.15, 369.13, 303.12, 167.07, 149.06, 123.04	209.65	As, SF1
174	36.75	Unknown	C25H35O10N	8.5	-4.49	510.2316	$[M+H]^+$: 482.20. 303.12, 167.07, 149.06, 123.04	171.14	As, SF1
175	36.76	Unknown	C23H31O10N	8.5	-4.77	482.2003	$[M+H]^+$: 465.17, 429.15, 303.12, 167.07, 149.06, 123.04	207.42	As, SF1

176		46.61	Unknown	C27H35O11N	10.5	-1.81	550.2273			$[M+H]^+$: 505.17, 343.12, 301.11, 286.08, 273.11, 269.08, 191.07, 167.07, 147.04	233.23	As, SF1
177		46.74	Unknown	C25H31O11N	10.5	-2.62	522.1962			$[M+H]^+$: 505.17, 467.27, 301.11, 167.07, 147.04	215.84	As, SF1
178		52.63	Unknown	C27H33O11N	11.5	-3.20	548.2114			$[M+H]^+$: 531.18, 301.11, 167.07, 147.04	186.63	As
179		55.21	Unknown	C34H47O13N	11.5	-4.50	678.3082			$[M+H]^+$: 625.26, 607.29, 411.14, 393.13, 303.12, 167.07	247.41	As
180		9.55	*(E)*-2-Hexenyl-*β*-sophorodide	C18H32O11	3.5	-0.34		423.1868	469.1925	$[M-H]^-$: 261.13, 221.07, 179.06, 161.05	200.59	Co, SF1
181		10.57	*(Z)*-3-Hexenyl-*β*-sophorodide	C18H32O11	3.5	-0.34		423.1864	469.1927	$[M-H]^-$: 261.13, 221.07, 179.06, 161.05	200.59	Co, SF1
182		11.26	Isomer of Lobetyolinin	C26H38O13	8.5	-0.60		557.2236	603.2291	$[M-H]^-$: 395.17, 233.12, 215.11, 179.06, 161.05	219.33	Co, SF1
183		12.40	(-)-*(7R,8S)*- dihydrodehydrodiconiferyl alcohol 4-O-*β*-D-glucopyranosyl-(1'''→2'')-*β*-D-glucopyranoside	C32H44O16	11.5	-0.77		683.2551	729.2606	$[M-H]^-$: 521.10, 341.10, 221.07, 179.06, 161.05	238.68	As, Co, SF1
184		12.74	*(E)*-2-hexenyl-*α*-L-arabinopyranosyl-(1→6)-*β*-D-glucopyranoside	C17H30O10	3.5	-0.59		393.1764	439.1819	$[M-H]^-$: 261.13, 217.12, 191.06, 179.06, 161.05	196.54	Co, SF1
185		13.12	Unknown	C18H34O11	2.5	0.39		425.2030	471.2085	$[M-H]^-$: 263.15, 245.09, 221.07, 179.06, 161.05	196.05	Co, SF1
186		14.19	Unknown	C18H34O11	2.5	-0.84		425.2025	471.2079	$[M-H]^-$: 263.15, 245.09, 221.07, 179.06, 161.05	196.05	Co, SF1
187	Polyacetylenes and polyenes	14.74	Lobetyolinin+Glu	C32H48O18	9.5	-0.75		719.2762	765.2830	$[M-H]^-$: 629.23, 485.15, 383.12, 341.11, 323.10, 221.07, 179.06, 161.05	256.31	Co, SF1
188		15.41	Pratialin B	C32H48O18	9.5	-0.75		719.2761	765.2825	$[M-H]^-$: 629.23, 485.15, 383.12, 341.11, 323.10, 221.07, 179.06, 161.05	256.31	Co, SF1
189		15.52	Unknown	C26H34O11	10.5	-0.37		521.2026	567.2082	$[M-H]^-$: 503.04, 341.14, 323.11, 197.08, 179.06, 161.05, 143.04	212.92	As, Co, SF1
190		15.68	Isomer of Lobetyolinin+Glu	C32H48O18	9.5	-0.85		719.2762	765.2824	$[M-H]^-$: 557.22, 377.16, 179.06, 161.05	256.31	Co, SF1
191		15.68	Unknown	C26H38O13	8.5	0.60		557.2243	603.2296	$[M-H]^-$: 539.21, 443.16, 377.21, 215.11, 197.10, 179.06, 161.05	219.33	Co, SF1
192		17.44	Codonopilodiynoside	C26H42O12	6.5	0.61		545.2607	591.2661	$[M-H]^-$: 527.20, 441.18, 361.22, 343.47, 179.07,	221.71	Co, SF1

								161.04, 149.05		
193	17.85	Isomer of Lobetyolinin	C26H38O13	8.5	-0.15	557.2239	603.2295	[M-H]-: 395.17, 377.16, 305.12, 233.12, 179.06, 161.05	219.33	Co, SF1
194	17.91	Unknown	C26H38O13	8.5	0.06	557.2240	603.2297	[M-H]-: 395.17, 377.16, 305.12, 233.12, 179.06, 161.05	219.33	Co, SF1
195	19.22	Isomer of Lobetyolinin	C26H38O13	8.5	0.28	557.2241	603.2299	[M-H]-: 233.12, 179.06, 161.05	219.33	Co, SF1
196	19.44	Unknown	C26H40O13	7.5	0.56	559.2399	605.2448	[M-H]-: 341.11, 323.10, 263.08, 217.12, 179.06, 161.05	239.72	Co, SF1
197	19.86	Unknown	C26H38O13	8.5	-0.15	557.2241	603.2298	[M-H]-: 443.16, 377.16, 305.12, 233.12, 179.06, 161.05	219.33	Co, SF1
198	20.72	*(3S,6E,9E)*-5-O-*β*-D-Glcp-(1→6)-O-*β*-D-Glcp-3,5,11-trihydroxy-3,7,11-trimethyldodeca-1,6,9-triene	C27H46O13	5.5	-2.33	577.2863	623.2918	[M-H]-: 414.99, 234.26, 215.01, 179.06, 161.05	239.13	Co, SF1
**199**	**21.12**	**Isomer of Lobetyolinin**	**C26H38O13**	**8.5**	**0.50**	**557.2242**	**603.2299**	**[M-H]-: 233.12, 179.06, 161.05**	**219.33**	**Co, SF1**
200	23.61	Cordifolioidyne C/Codonopilodiynoside A	C20H28O8	7.5	1.08	395.1711	441.1765	[M-H]-: 305.13, 233.12, 215.11, 179.06, 143.07	194.25	Co, SF1
201	24.21	Isomer of Cordifolioidyne C/Codonopilodiynoside A	C20H28O8	7.5	1.54	395.1712	441.1766	[M-H]-: 305.12, 215.11, 179.05, 143.07	194.25	Co, SF1
202	25.28	Codonopiloenynenoside A	C20H30O8	6.5	-0.36	397.1866	443.1921	[M-H]-: 235.13, 217.12, 179.06, 161.05	185.22	Co, SF1
**203**	**25.32**	**Lobetyolin**	**C20H28O8**	**7.5**	**0.93**	**395.1715**	**441.1770**	**[M-H]-: 305.12, 233.12, 215.11, 185.10, 143.07**	**194.25**	**Co, SF1**
204	25.55	Cordifolioidyne C/Codonopilodiynoside A/ Codonopilodiynoside L/Codonopilodiynoside M	C20H28O8	7.5	2.32	395.1715	441.1771	[M-H]-: 305.12, 215.11, 179.06, 143.07	194.25	Co, SF1
205	30.21	Unknown	C20H36O12	3.5	-0.49	467.2132	513.2186	[M-H]-: 425.20, 407.18, 305.20, 263.15, 221.07, 179.06, 161.05	200	As, Co, SF1
206	30.51	Unknown	C32H52O16	7.5	1.56	691.3193	737.3241	[M-H]-: 529.27, 397.22, 293.09, 221.07, 179.06, 161.05	229.57	Co
207	32.77	Codonopilodiynoside G	C26H38O12	8.5	0.70	541.2294	587.2346	[M-H]-: 341.11, 323.10, 263.08, 217.12, 179.06, 161.05	237.16	Co, SF1
208	38.32	Unknown	C27H46O12	5.5	0.82	561.2916	607.2971	[M-H]-: 399.24, 341.20, 221.07, 179.06, 161.05	225.67	Co

209		40.57	Codonopilodiynoside G/Kodonopilodiynoside K	$C_{26}H_{38}O_{12}$	8.5	0.81	541.2289	587.2344	[M-H]$^-$: 341.11, 323.10, 263.08, 217.12, 179.06, 161.05	214.95	Co, SF1
210		11.78	Tangshenoside I	$C_{29}H_{42}O_{18}$	9.5	-0.60	677.2294		[M-H]$^-$: 497.17, 453.18, 261.10	256.9	Co
211		14.98	Tangshenoside Ⅴ	$C_{21}H_{26}O_{12}$	9.5	-1.02	469.1347		[M-H]$^-$: 325.09, 265.07, 235.06, 163.04, 143.04	213.52	Co, SF1
212		16.47	Hexyl-1-O-*α*-L-arabinofuranosyl(1→6)-*β*-D-glucopyranoside	$C_{17}H_{32}O_{10}$	2.5	0.13	395.1923	441.1978	[M-H]$^-$: 263.15, 233.07, 191.06, 161.05	194.25	Co, SF1
213		17.81	Isomer of Hexyl-1-O-*α*-L-arabinofuranosyl(1→6)-*β*-D-glucopyranoside	$C_{17}H_{32}O_{10}$	2.5	-0.41	395.1921	441.1979	[M-H]$^-$: 263.15, 233.07, 191.06, 161.05	194.25	Co, SF1
**214**		**21.11**	**Syringaresinol 4'-O-glucopyranoside**	**$C_{28}H_{35}O_{13}$**	**11.5**	**1.79**	**579.2094**	**625.2141**	**[M-H]$^-$: 417.16, 402.13, 387.10, 371.15, 181.05**	**209.73**	**As, SF1**
215		28.35	Codonoside A	$C_{38}H_{48}O_{20}$	15.5	0.21	823.2657		[M-H]$^-$: 689.33, 575.27, 539.2565	283.35	Co
216		45.92	9,12,13-Trihydroxy-10,15-octadecadienoic Acid	$C_{18}H_{32}O_5$	3.5	0.13	327.2177		[M-H]$^-$: 309.16, 291.18, 229.12, 211.11, 171.10	177.48	As, Co, SF1
217	**Other**	49.71	9,12,13-Trihydroxy-10-octadecadienoic Acid	$C_{18}H_{34}O_5$	2.5	-1.25	329.2337		[M-H]$^-$: 311.19, 293.21, 229.12, 211.10, 171.10	175.19	As, Co, SF1
218		50.17	9,12,13-Trihydroxy-11-octadecadienoic Acid	$C_{18}H_{34}O_5$	2.5	1.07	329.2334		[M-H]$^-$: 311.18, 293.22, 229.11, 211.11	175.19	As, Co, SF1
219		50.69	9,12,13-Trihydroxy-12-octadecadienoic Acid	$C_{18}H_{34}O_5$	2.5	-0.02	329.2333		[M-H]$^-$: 311.18, 293.21, 171.04	175.19	As, Co, SF1
220		55.86	Unknown	$C_{30}H_{54}O_{10}$	4.5	-0.39	573.3642	619.3347	[M-H]$^-$: 555.35, 499.33, 481.32, 329.23, 293.21, 211.13, 171.10	225.97	As, Co
221		57.52	9,10-Dihydroxy-12-Octadecenoic Acid-2H	$C_{18}H_{32}O_4$	3.5	-0.49	311.2226		[M-H]$^-$: 293.22, 275.22, 201.09, 181.13	175.58	As, Co, SF1
222		58.38	9,10-Dihydroxy-12-Octadecenoic Acid	$C_{18}H_{34}O_4$	2.5	0.69	313.2386		[M-H]$^-$: 295.23, 277.22, 195.12, 183.12	175.53	As, Co
223		58.76	Isomer of 9,10-Dihydroxy-12-Octadecenoic Acid	$C_{18}H_{34}O_4$	2.5	0.18	313.2385		[M-H]$^-$: 295.23, 201.11, 171.10	175.53	As, Co

Bold indicates the components were compared with reference compounds.
